# Comparative physical maps derived from BAC end sequences of tilapia (*Oreochromis niloticus*)

**DOI:** 10.1186/1471-2164-11-636

**Published:** 2010-11-16

**Authors:** Lucile Soler, Matthew A Conte, Takayuki Katagiri, Aimee E Howe, Bo-Young Lee, Chris Amemiya, Andrew Stuart, Carole Dossat, Julie Poulain, Jeremy Johnson, Federica Di Palma, Kerstin Lindblad-Toh, Jean-Francois Baroiller, Helena D'Cotta, Catherine Ozouf-Costaz, Thomas D Kocher

**Affiliations:** 1 CIRAD-PERSYST, Aquaculture Research Unit, TA B-20/A, Campus International de Baillarguet, 34398 Montpellier cedex 5, France; 2 Department of Biology, University of Maryland, College Park, Maryland 20742, USA; 3 Laboratory of Fish Health Management, Tokyo University of Marine Science and Technology, 4-5-7 Konan, Minato-Ku Tokyo, 108-8477, Japan; 4 Genome Resource Center, Benaroya Research Institute at Virginia Mason, 1201 Ninth Avenue, Seattle, WA 98101 USA; 5 CEA, DSV, Genoscope, 2 rue Gaston Crémieux, CP5706 91057 Evry cedex, France; 6 Broad Institute, 7 Cambridge Center, Cambridge, Massachusetts 02142, USA; 7 CNRS UMR 7138 « Systématique, Evolution, Adaptation », MNHN Département Systématique et Evolution, C.P. 26, 57 rue Cuvier 75231 PARIS Cedex 05, France

## Abstract

**Background:**

The Nile tilapia is the second most important fish in aquaculture. It is an excellent laboratory model, and is closely related to the African lake cichlids famous for their rapid rates of speciation. A suite of genomic resources has been developed for this species, including genetic maps and ESTs. Here we analyze BAC end-sequences to develop comparative physical maps, and estimate the number of genome rearrangements, between tilapia and other model fish species.

**Results:**

We obtained sequence from one or both ends of 106,259 tilapia BACs. BLAST analysis against the genome assemblies of stickleback, medaka and pufferfish allowed identification of homologies for approximately 25,000 BACs for each species. We calculate that rearrangement breakpoints between tilapia and these species occur about every 3 Mb across the genome. Analysis of 35,000 clones previously assembled into contigs by restriction fingerprints allowed identification of longer-range syntenies.

**Conclusions:**

Our data suggest that chromosomal evolution in recent teleosts is dominated by alternate loss of gene duplicates, and by intra-chromosomal rearrangements (~one per million years). These physical maps are a useful resource for comparative positional cloning of traits in cichlid fishes. The paired BAC end sequences from these clones will be an important resource for scaffolding forthcoming shotgun sequence assemblies of the tilapia genome.

## Background

Tilapia (*Oreochromis *spp.) are among the most important species in aquaculture and a primary source of animal protein for millions of people in the developing world [1]. Only limited efforts have been made toward genetic improvement of these species [2]. The sequence of the tilapia genome will be a fundamental resource used for genetic selection, on traits such as growth performance and disease resistance, to create strains of fish optimized for the unique culture conditions of each country.

Tilapia and other closely related species of African cichlid fishes are also widely used in basic research. Because of their intimate physiological relationship with the environment, tilapia are ideal for studies of ion regulation [3,4], the accumulation of heavy metals [5], and detoxification of biotoxins [6]. Nile tilapia expressing a humanized insulin gene are being studied as a source of islet cells which might be transplanted into humans for control of type I diabetes [7]. Tilapia are also an important model for studying environmental influences on sex differentiation [8]. The closely related haplochromine cichlids of the East African lakes are a model system for studying the genetic basis of behavior [9] and evolutionary processes of adaptation and speciation [10].

### Cichlid genomics

Considerable progress has been made in developing genomic resources for tilapia and other East African cichlid fishes. Genetic maps have been published for tilapia [11], Lake Malawi haplochromines [12], and *Astatotilapia burtoni *[13]. There are also extensive collections of ESTs for Lake Victoria haplochromines [14,15], *A. burtoni *[16,17] and Nile tilapia [18]. Several BAC libraries have been constructed for Nile tilapia [19], and fingerprinted to construct a physical map [20]. BAC libraries have been constructed also for haplochromine cichlids from lakes Malawi [21], Victoria [22] and Tanganyika [23].

### Comparative physical maps

Comparative maps have been a useful intermediate resource for many agricultural species before complete genome sequences were available [24-26]. Most often these comparative maps have relied on mapping homologous gene markers in radiation hybrid panels [27], but comparative maps have also been based on analysis of BAC end sequences [28,29]. Until a complete genome sequence is available for tilapia, comparative maps to the genome sequences of model fish species will provide the best organization of the partial sequence data for cichlid fishes.

The utility of a comparative map is proportional to the extent to which synteny exists between the two genomes. Useful comparative maps have been constructed between cattle and human (100MY divergence)[30]. The divergence among many fish lineages is much older, creating the potential for more extensive genome rearrangements. The Ostariophysi (e.g. zebrafish) and Acanthopterygii (e.g. medaka) diverged ~300MY ago [31]. Divergence among Percomorph groups (e.g. Tilapia and pufferfish) occurred more than 100MY ago [32]. The utility of comparative maps across these greater evolutionary distances is not yet clear.

Early research suggested that the rate of chromosome evolution is relatively low in non-mammalian vertebrates [33]. Recently it has been suggested that the rate of chromosomal rearrangement increases immediately after episodes of whole-genome duplication [34]. Teleost fishes experienced an additional round of whole genome duplication about 300 MY ago [35], and recent papers have suggested that fishes continue to have a high rate of chromosomal rearrangement [36]. However, the more extensive inter-chromosomal rearrangements detected in the zebrafish genome may be due to unique evolutionary processes in that lineage, and there appear to have been no major inter-chromosomal rearrangements in the medaka genome during the last 300MY [37]. The green pufferfish shows relatively little inter-chromosomal rearrangement since divergence from the ancestral bony vertebrate [38]. Most of the changes in the pufferfish lineage represent fusions that reduced the chromosome number after whole genome duplications.

The goal of the present study was to construct a comparative physical map between tilapia and the latest sequence assemblies for three other percomorph species: stickleback, medaka and pufferfish. From this comparative map we estimate the extent of chromosomal rearrangement during the recent evolution of these species.

## Results and Discussion

### New BAC library

The BAC library (VMRC-44) constructed at the Benaroya Research Institute consists of 73,728 clones (192 384-well plates) with an average insert size of 150 kb. This represents a total of 11 Gbp or approximately 10× coverage of the tilapia genome. The methods used to prepare this library are presented in Additional file 1.

### Sequencing statistics

#### Genoscope

The construction of the BAC libraries sequenced at Genoscope was reported previously [19]. A total of 35,000 clones from these libraries (average insert 182 kb, ~5.6× genome coverage), have been restriction fingerprinted and assembled into 3,600 contigs [20]. Genoscope end sequenced a total of 40,704 clones (52 plates from library 3 and 54 plates from library 4). From 37,383 clones, a total of 68,032 end sequences were obtained, representing 6.8× clone coverage of the genome. The mean trimmed length of the sequences was 562.6 bp, for a total dataset of 38,272,386 bp representing 3.8% sequence coverage of the genome.

#### Broad

The Broad Institute end sequenced 73,728 clones (192 plates) from the Benaroya library, obtaining a sequence for at least one end of 68,876 clones, representing 10.0× clone coverage of the genome. Multiple attempts were made to sequence some clones and therefore, a total of 153,216 end sequences were finally submitted to GenBank. The mean length of the sequences was 757.3 bp, for a dataset of 116,029,366 bp. After quality trimming and vector removal with Lucy, a total of 124,995 sequences remained, with a mean length of 527.3 bp, for a total of 65,912,624 bp, representing 6.6% sequence coverage of the genome. These sequences were previously analyzed for their repeat content [39].

### Microsatellites

Microsatellite motifs were identified in 7,230 (3.7%) of the 193,027 sequences. These included 5,027 dinucleotide, 1,250 trinucleotide, and 953 tetranucleotide repeats (Additional file 2**Table S1**). Over half of the repeats (3,887) were AC dinucleotides. AT and AG dinucleotides were also abundant. AAT was the most frequent trinucleotide. These microsatellites could be exploited to develop new genetic markers and could be used to anchor the FPC-based physical map [20] to the genetic map [11].

### Genes

A total of 16,636 (8.6%) repeat-masked sequences had a significant (1e-5) BLASTx hit to the Uniprot database. We found that 38,020 (19.7%) of the repeat-masked sequences had a significant (1e-50) BLASTn hit to the 116,899 Nile tilapia EST set [40]. Therefore, 49,823 (25.8%) of the sequences had either a significant BLASTx hit to Uniprot or a significant BLASTn hit to the Nile tilapia ESTs. There were 4,833 (2.5%) sequences that had a significant hit to both Uniprot and the Nile tilapia ESTs.

### Comparative mapping

A total of 193,027 BAC end sequences were BLASTed against the genome assemblies of stickleback, medaka and pufferfish. The results are summarized in Table 1. The proportion of sequences that had hits with e-values less than e^-10 ^ranged from 11 percent against pufferfish, 15 percent against medaka and 17 percent against stickleback. Twenty-eight percent of the BACs had at least one hit to the stickleback genome assembly.

**Table 1:** BLAST statistics against three fish genome assemblies.

	# BACs one end	# BACs both ends	Sequences w/hit	BACs w/hit	Type 1*	Type 2*	Type 3*	Type 4*	3/(2+3)	(3+4)/(2+3+4)
**Stickleback**										

Genoscope	6,734	30,649	11,229	10,048	7,987	797	54	153	0.063	0.206

Broad	12,758	56,118	21,754	19,510	14,405	1,416	142	633	0.054	0.286

Combined	19,492	86,767	33,053	29,558	22,392	2,213	196	786	0.057	0.259

										

**Medaka**										

Genoscope	6,734	30,649	9,764	9,278	7,087	469	43	226	0.084	0.364

Broad	12,758	56,118	19,699	17,943	13,907	886	51	624	0.054	0.432

Combined	19,492	86,767	29,463	27,221	20,994	1,355	94	850	0.065	0.410

										

**Tetraodon**										

Genoscope	6,734	30,649	6,931	6,386	3,879	265	14	62	0.050	0.222

Broad	12,758	56,118	14,260	13,227	7,279	503	19	188	0.036	0.291

Combined	19,492	86,767	21,191	19,613	11,158	768	33	250	0.041	0.269


*Numbers for type 1, 2, 3 & 4 do not include hits to the contigs in the 'unordered chromosome' of each genome assembly. Type 2 hits were scored when the two end sequences of a clone hit within 300 kb of each other in the target genome.

We classified the BACs into one of four types, according to the pattern of BLAST hit. Type 1 clones are those for which only a single sequence produced a hit in the target genome. Type 2 clones are those in which the sequences from the two ends of the BAC hit in the appropriate opposing orientation within 300 kb in the target genome. Type 3 clones are those in which the two end sequences of a BAC hit the same chromosome in the target genome outside of the 300 kb range. Type 4 BACs are those in which the two sequences hit different chromosomes in the target genome.

Since the average BLAST hit rate against the stickleback genome is 17%, we expected the proportion of clones with hits on both ends would be 2.9%. In fact we observed a slightly greater proportion (3.7%), possibly reflecting a clustering of conserved sequences in the genomes. When both ends of a BAC had BLAST hits, they were most often found within 300 kb on the same chromosome in the target genome (type 2). A much smaller proportion (3-5%) were found at larger distances on the same chromosome in the target genome (type 3).

### Conservation of gene order

We can use the ratios of type 2, 3 and 4 hits (Table 1) to estimate the number of rearrangements between genomes. Across the three species, 27-41% of double hit clones are type 3 or 4. If the BAC clone inserts average 150 kb, and every third clone has a break in synteny, it would suggest a breakpoint every 3 × 150 kb = 450 kb across the genome. This is equivalent to more than 2000 breakpoints across the genome, or about 100 breakpoints per chromosome. We suspect this simple statistic overestimates the true number of chromosomal rearrangements.

The best estimate of intra-chromosomal rearrangements is the number of type 3 BACs relative to the number of type 2 + type 3 BACs. This proportion is between 3 and 6%, suggesting an intra-chromosomal rearrangement every 20 × 150 kb = 3 Mb. If the average chromosome is 48 Mb, this suggests about 16 breakpoints (e.g. 8 inversions) per chromosome. We detected a mean of 2.1 breakpoints per chromosome, with at least one rearrangement on each stickleback chromosome (Additional file 3**Table S2**). The observed breakpoints were spanned by an average of 3.5 BAC clones. Unfortunately, the relatively low clone coverage of the type 3 BACs does not allow us to identify all of the likely breakpoints, or precisely map their locations. Still, the high end of these estimates (8 inversions/chromosome) suggests there have been only 160 inversions since the divergence of tilapia and stickleback. The type 3 hits are visualized in Circos plots in Additional files 4, 5, 6, **Figures S1-S3**.

**Figure 1:**
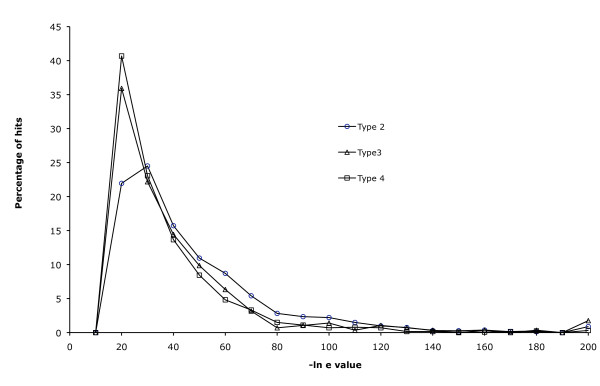
**Distribution of BLAST scores for type 2, 3 and 4 hits of the Broad Institute tilapia BAC end sequences on the stickleback genome assembly**. The distribution was truncated at e^-10^.

Type 4 BACs are possible evidence of inter-chromosomal rearrangements, and represent 24-37% of the two-hit BACs. This might suggest more than 100 breakpoints in synteny for each chromosome. However, we do not think this statistic is an indication of a large number of inter-chromosomal transfers of genes. Rather, it probably includes many instances in which one of the BLAST matches is to a paralog on a second chromosome. For example, if the syntenic copy of the gene has been lost, BLAST will identify a paralog on another chromosome as the best hit. This kind of gene loss is a common feature of fish genomes, which underwent a whole-genome duplication about 300MY ago. Alternate loss of even a small proportion of genes from these duplicated regions would be sufficient to create the pattern. There are about 1,250 genes/chromosome, and if only 5% of them (60 genes/chromosome) were deleted after the whole genome duplication, it would be sufficient to create the pattern we see in the BAC data. The fact that type 4 BLAST hits have much lower e-values than type 2 BLAST hits (Figure 1) tends to reinforce this view.

We mapped the rearrangements onto a phylogeny of the four species. The results suggest that approximately 15-20 rearrangements have occurred on each lineage since they diverged from their common ancestor. There is no indication that the rate of rearrangement is higher in one lineage than another.

### Comparative physical maps

**Figure 2:**
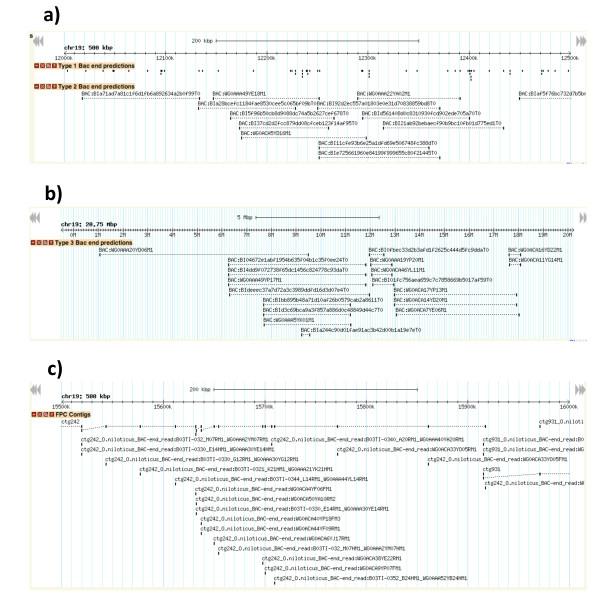
**Comparative mapping of tilapia data mapped on the stickleback genome assembly displayed in GBrowse at http://www.BouillaBase.org**. a) Type 1 and 2 BLAST hits, b) Type 3 BLAST hits, c) FPC contigs.

These BLAST results are displayed in a GBrowse interface at http://www.BouillaBase.org (Figure 2). Separate tracks display the type 1, 2, 3 and 4 BLAST hits. An additional track displays the BLAST hits from each of the fingerprint contigs in the previous physical map [20]. Because these FPC contigs contain multiple BAC clones, they help to tie the physical map together at larger scales than the end sequences of individual clones.

## Conclusions

End-sequencing of these BAC libraries was a key step in preparing the tilapia genome for shotgun sequencing. Together with the BAC fingerprint database, these sequences will provide long-range structure for scaffolding the contigs of genome assemblies to construct a golden path across the genome.

Recent molecular phylogenies appear to have reached a consensus that cichlids are more closely related to medaka than to either pufferfish or stickleback [40-42]. Nevertheless, a higher number of the tilapia BAC end sequences hit stickleback (33,053) than either medaka (29,463) or pufferfish (21,191). This discrepancy might be due to variation in the quality of each assembly, or it might support an alternative phylogenetic reconstruction. Regardless, it appears that the stickleback sequence is currently the best reference sequence for building comparative maps of tilapia [43].

Finally, these data suggest that chromosomal evolution in recent teleosts is dominated by alternate loss of gene duplicates, and by intra-chromosomal rearrangements. The rate of these rearrangements is relatively slow, on the order of one per million years. So the prospects are good for building useful comparative maps between sequenced genomes and the large number of as yet unsequenced teleost species of commercial or scientific importance.

## Methods

### Sequence trimming

Both trimmed and untrimmed quality scores and FASTA sequences for the Genoscope library 4 sequences were available, whereas only trimmed FASTA sequences for the Genoscope library 3 were available. To achieve essentially the same level of trimming for both Genoscope libraries and the Broad library, the Genoscope library 4 data was used to determine a set of parameters that trimmed the data in the same way as had been done for both the Genoscope libraries. The following Lucy 1.20p [44] settings were used: -*error 0.025 0.02*, -*bracket 10 0.005*, -*window 50 0.08 10 0.12*, and -*vector *with the FASTA sequence of the pBAC-Lac cloning vector [45] for the Genoscope libraries and the FASTA sequence of the pCC1BAC cloning vector (Epicentre Biotechnologies) for the Broad library.

### Annotation

#### Identification of microsatellites

We scanned the BAC end sequences for microsatellites that might be useful for genetic mapping. We used the Tandem Repeats Finder http://tandem.bu.edu/trf/trf.html[46] to identify microsatellite motifs. The BAC ends containing microsatellites have been color-coded in the annotation tracks in the GMOD browser.

#### Identification of genes

The BAC end sequences were masked with RepeatMasker version open-3.2.8 [47] against a combination of the Repbase [48] RepeatMasker libraries, release 20090604 and tilapia specific repeats [39]. The sequences were then aligned to the Uniprot database (release-2010_05) using BLASTx, and a database of 116,899 Sanger ESTs from Nile tilapia [18] using BLASTn. Significant hits were defined with an e-value threshold of 1e^-5 ^for Uniprot, or 1e^-50 ^for the ESTs.

### BLAST analysis

Comparative mapping was performed by running BLASTn against the pufferfish, stickleback and medaka genome assemblies. The genomes were downloaded from the UCSC Genome Browser http://hgdownload.cse.ucsc.edu/downloads.html. The following versions were used for the respective genomes: Feb. 2004 (Genoscope 7/tetNig1), Feb. 2006 (Broad/gasAcu1), and Oct. 2005 (NIG/UT MEDAKA1/oryLat2). FASTA sequences were downloaded and formatted into BLAST databases for use with the NCBI BLASTall tool and scripts utilizing BioPerl were used to parse the results. Type 2 hits were defined as mate pairs that hit the target genome in opposing orientation at a distance of 300 kb or less. Type 3 hits were defined as mate pairs that hit the same chromosome, regardless of orientation. Type 4 hits were defined as mate pairs that hit different chromosomes. The positions of the BLAST hits were visualized with Circos [49].

### Online access to the resource

We used the GMOD browser http://www.gmod.org to develop a comparative genome server for fishes that maps tilapia ESTs and BAC end-sequences onto the genome assemblies of stickleback, medaka and pufferfish. This server can be accessed through our www site http://www.BouillaBase.org.

## Additional data files

The Benaroya/Broad BAC end sequences are available in the NCBI Trace Archive under Center_Project 'G1447'. The Genoscope sequences are available as accession numbers FQ242537 - FQ280267.

## Authors' contributions

LS and MAC carried out the bioinformatic analyses. TK, AEH and BYL constructed and prepared the BAC libraries for sequencing at Genoscope. CA and AS constructed the BAC library that was sequenced at the Broad Institute. CD and JP sequenced the BAC libraries at Genoscope. JJ, FDP and KLT organized the sequencing at the Broad Institute. JFB, HDC, COC and TDK prepared the manuscript. All authors read and approved the final manuscript.

## Supplementary Material

Additional file 1 **Supplemental Methods**. Description of methods used in constructing the BAC library. Click here for file

Additional file 2 **Table S1 Microsatellite motifs identified in the BAC end sequences**. Click here for file

Additional file 3 **Table S2 Number of type 3 BACs spanning potential recombination breakpoints in the comparative map to stickleback**. Click here for file

Additional file 4 **Figure S1 Circos plot of the type 3 BLAST hits on the stickleback genome**. The chromosomes of the stickleback genome are represented on the circle. The position of BAC mate pair BLAST hits are indicated with red arcs. Click here for file

Additional file 5 **Figure S2 Circos plot of the type 3 BLAST hits on the medaka genome**. The chromosomes of the medaka genome are represented on the circle. The position of BAC mate pair BLAST hits are indicated with red arcs. Click here for file

Additional file 6 **Figure S3 Circos plot of the type 3 BLAST hits on the *Tetraodon *genome**. The chromosomes of the *Tetraodon *genome are represented on the circle. The position of BAC mate pair BLAST hits are indicated with red arcs. Click here for file
