# Supplementary material for: Comparative physical maps derived from BAC end sequences of tilapia (Oreochromis niloticus)
Source: BMC Genomics. 2010 Nov 16;11:636. doi: 10.1186/1471-2164-11-636 (PMC3018143; doi:10.1186/1471-2164-11-636)
Supplement: Additional file 1 — Supplemental Methods. Description of methods used in constructing the BAC library. [file 1471-2164-11-636-S1.PDF]

SUPPLEMENTAL METHODS

BAC library construction

Construction of the BAC libraries sequenced at Genoscope has been described previously [19]. The library sequenced at the Broad Institute was constructed as follows:

Embedding of high molecular weight (HWM) DNA: A single tilapia (Scotland, ID 000638D3DF) was used to make 55 HWM DNA blocks. Blood was obtained from the heart and placed into 5ml 1X PBS with 50mM EDTA and centrifuged for 5 min. at 2,000 rpm (Sigma 4K15C centrifuge, rotor Nr. 12170-H). The supernatant was removed and the pellet resuspended in 2ml 1X PBS with 50mM EDTA. A 1:20 dilution in 1ml volume was performed for cell counting and it was determined that the suspension contained 964 ug DNA. Next the suspension was equilibrated at 37°C for 10 min. 1ml of resuspended solution was added into 1ml 2% Incert agarose (in homogenization buffer). Each well of a plug mold (BioRad) was filled with 80ul suspension and repeated until all solution was used. The molding was placed at 4°C for 10 min. to ensure plugs were completely solidified. Plugs were then treated with cell lysis solution (1% LDS, 10mM Tris pH 8.0, 150mM EDTA pH 8.0) at 37°C for 36 hours. Cell lysis solution was changed two times over the course of the incubation period. Finally, plugs were transferred to storage solution (0.2% N-laurylsarcosine, 2mM Tris pH 8.0, 140mM EDTA pH 8.0) and stored at 4°C.

Size fragmentation: Genomic DNA (1/2 plug per reaction) was partially digested at 37° C for 2.5 hours in 500ul reaction volumes containing 6 units of the restriction enzyme EcoRI (New England Biolabs) and 60 units EcoRI methylase. The quantity of EcoRI and EcoRI methylase were optimally obtained from titration experiments using

various amounts of EcoRI and EcoRI methylase on 1/6th plug per 500ul reaction. Other components of the reaction were 2.6mM spermidine, 0.5mg/ml BSA (New England Biolabs) and 1x EcoRI reaction buffer (0.08mM S-adenosylmethionine, 2mM $MgCl_2$, 1mM DTT). Following the incubation period, the reaction was stopped by adding 30ul Proteinase K (10mg/ml), 37.5ul 20% NDS, 150ul 0.5M EDTA, and incubated at 37° C for 1 hour. Proteinase K activity was eliminated by transferring plugs into 15ml ½X TE and 15ul 100mM PMSF using 15ml falcon tubes. Incubation at 4°C for 20 min., repeated 3 times. Next, the plugs were equilibrated in 50ml ½X TE for 1 hour at 4°C. DNA fragments were separated on a 1% agarose gel (Pulse-Field certified Bio Rad) using pulse-field-gel-electrophoresis (BioRad CHEF XA Mapper) in ½X TBE buffer. A 24 hour, multi-state "trash run" was used before the preparative run in order to remove unwanted, smaller-sized DNA molecules. This run contains six separate timed "blocks," with blocks 1, 3, 5 and blocks 2, 4, 6 being identical to each another. The conditions for blocks 1, 3, 5 are as follows: 5 V/cm, 4 hours, 15 sec. initial time, 15 sec. final time, 14°C, 120° reorientation angle (120° and -120°). The conditions for blocks 2, 4, 6 are the same except for the following: 120° reorientation angle (60° and -60°). Before beginning the trash run, the plugs were loaded into the large preparative well and low range PFG markers (New England BioLabs) were loaded into the outermost wells. Upon completion of the trash run, fresh buffer and low range PFG markers were added before beginning the preparative run. The preparative run was performed at the following conditions: 30 hours, 10 sec. initial time, 60 sec. final time, field angle120°, 5 V/cm, 14°C.

Size Selection: Gel fragments were excised from the preparative lane that contained the HWM DNA fragments. Eight fractions ranging from 50kb-300kb were

removed from the agarose. A sliver from each fragment was used in a step ladder gel to determine the size range of the DNA fragments. Fractions 4 and 5 were chosen for electroelution and were equilibrated in ½X TBE. DNA fragments were electroeluted at 4 V/cm for 4 hours at 4°C and then dialyzed O/N in 4L ½X TE at 4°C. A 0.8% agarose gel was used to determine the quantity of DNA recovered from the electroelution.

Ligation: A CopyControl BAC Cloning Kit (Epicentre) was used for the ligation of the EcoRI-digested HMW DNA fragments. Ligation reactions (50ul volume) consisted of ~100ng DNA, 25ng CopyControl pCC1BAC (EcoRI) vector, 1X Fast-link Ligation Buffer, 1mM ATP, and 2U Fast-link DNA Ligase (2U/ul). (Note: after combining the HWM DNA and vector DNA, the reaction was heated at 55°C for 10 minutes. The remaining ligation components were added 10 minutes later once the samples had cooled to room temperature). Ligation reactions were incubated overnight at 16°C. The reactions were stopped by adding 1ul 0.5M EDTA, 1ul 10mg/ml Proteinase K, and incubated for 1 hour at 37°C. To eliminate Proteinase K activity, 1ul 100mM PMSF was added to each reaction and incubated 1 hour at room temperature. To remove salts, the ligation reactions were drop dialyzed on 0.025um nitrocellulose filters (Millipore) for 1.5 hours against ddH20 at 4°C. The sample volume was then reduced to ~20ul by drop dialyzing against 30% PEG (PEG8000) in ½X TE at 4°C.

Transformation: Using a BTX ECM 630, 10ul DNA ligation was added to 100ul DH10B T1 resistant electrocompetent cells (Invitrogen). Electroporations were carried out in 2mm cuvettes at conditions of 2.5kV, 225Ohms, 25uF. Transformed cells were allowed to recover in 50ml conical tubes, each containing 10ml SOC (2 transformations per tube) and incubated for 1 hour at 37°C, 250 rpm. Next, 1ml 100% glycerol was

added to each 50ml tube, thoroughly mixed, and 100ul of transformants were plated on LB/CAM/IPTG/X-Gal plates and incubated overnight at 37°C. The remaining transformants were flash-frozen using liquid nitrogen and stored at -80°C until time of plating.

Insert Size screening: An initial screening of clones was performed using Epilyse (Epicentre) on 48 random white colonies (24 per fraction), which determines the frequency of inserts and a rough estimate of size. For further analysis, DNA from 40 clones were isolated using a standard alkaline lysis miniprep procedure and resuspend in 30ul ddH20. Each clone was digested using NotI (15ul reaction volume, incubated 2 hours at 37°C) and sizing was accomplished using pulse-field-gel-electrophoresis (15 hours, 1 sec. initial time, 20 sec. final time, 14° C, field angle120°, 6 V/cm) with the low range PFG marker.

Library Array: Transformants that contained average insert size of 150kb were plated on LB/CAM/IPTG/X-GAL plates (incubated overnight at 37°C) and picked into 384-well LB/CAM/glycerin micro-titer plates (Genetix) using a colony-picking robot (Norgren Systems). A total of 192 plates were picked for this particular library. A Total Array System (*Bio*Robotics) machine was used for generating high-density nylon filter sets (22cm x 22cm) containing BAC DNA. In addition, the Total Array System was also used for 2 copies of this library (copy 1 with Genetix plates, copy 2 with Greiner plates).
