# Supplementary material for: Comparative physical maps derived from BAC end sequences of tilapia (Oreochromis niloticus)
Source: BMC Genomics. 2010 Nov 16;11:636. doi: 10.1186/1471-2164-11-636 (PMC3018143; doi:10.1186/1471-2164-11-636)
Supplement: Additional file 2 — Table S1 Microsatellite motifs identified in the BAC end sequences. [file 1471-2164-11-636-S2.PDF]

Table S1. Microsatellite motifs identified in the BAC end sequences.

Sequence	Genoscope	Broad	Combined
AC	1567	2320	3887
AAT	414	469	883
AT	290	489	779
AG	101	260	361
ATCC	66	137	203
AGAT	51	123	174
AAAT	53	84	137
AAAC	42	85	127
AAG	35	48	83
ATC	26	56	82
AAC	23	43	66
AAAG	19	41	60
AGG	21	39	60
AATC	18	39	57
ACAG	13	26	39
AGC	16	22	38
ACT	13	20	33
ACAT	7	20	27
AAGG	11	11	22
AATG	5	17	22
ACTC	4	13	17
AAGT	5	9	14
AGCG	1	9	10
AATT	0	8	8
ACTG	0	7	7
ACGC	6	0	6
AGGG	1	5	6
ACCT	4	0	4
AGCT	3	1	4
AACT	3	0	3
ACC	2	1	3
AACC	1	1	2
AGGC	2	0	2
AAGC	1	0	1
ACG	1	0	1
ACGG	0	1	1
CCG	1	0	1
**Totals**	**2826**	**4404**	**7230**
