# Supplementary material for: Comparative physical maps derived from BAC end sequences of tilapia (Oreochromis niloticus)
Source: BMC Genomics. 2010 Nov 16;11:636. doi: 10.1186/1471-2164-11-636 (PMC3018143; doi:10.1186/1471-2164-11-636)
Supplement: Additional file 3 — Table S2 Number of type 3 BACs spanning potential recombination breakpoints in the comparative map to stickleback. [file 1471-2164-11-636-S3.PDF]

Table S2. Number of type 3 BACs spanning potential recombination breakpoints in the comparative map to stickleback.

Stickleback chromosome	Number of rearrangements	# of clones spanning each rearrangement
1	4	3,4,2,2
2	2	4,4
3	2	5,1
4	2	2,2
5	0	
6	2	1,1
7	3	5,4,1
8	3	10,6,5
9	3	2,5,2
10	2	1,2
11	3	1,1,6
12	3	8,8,5
13	1	3
14	3	1,2,2
15	2	4,7
16	1	3
17	1	6
18	1	5
19	3	7,4,5
20	3	2,1,2
21	1	1
