# Supplementary figures and images for: Comparative physical maps derived from BAC end sequences of tilapia (Oreochromis niloticus)
Source: BMC Genomics. 2010 Nov 16;11:636. doi: 10.1186/1471-2164-11-636 (PMC3018143; doi:10.1186/1471-2164-11-636)

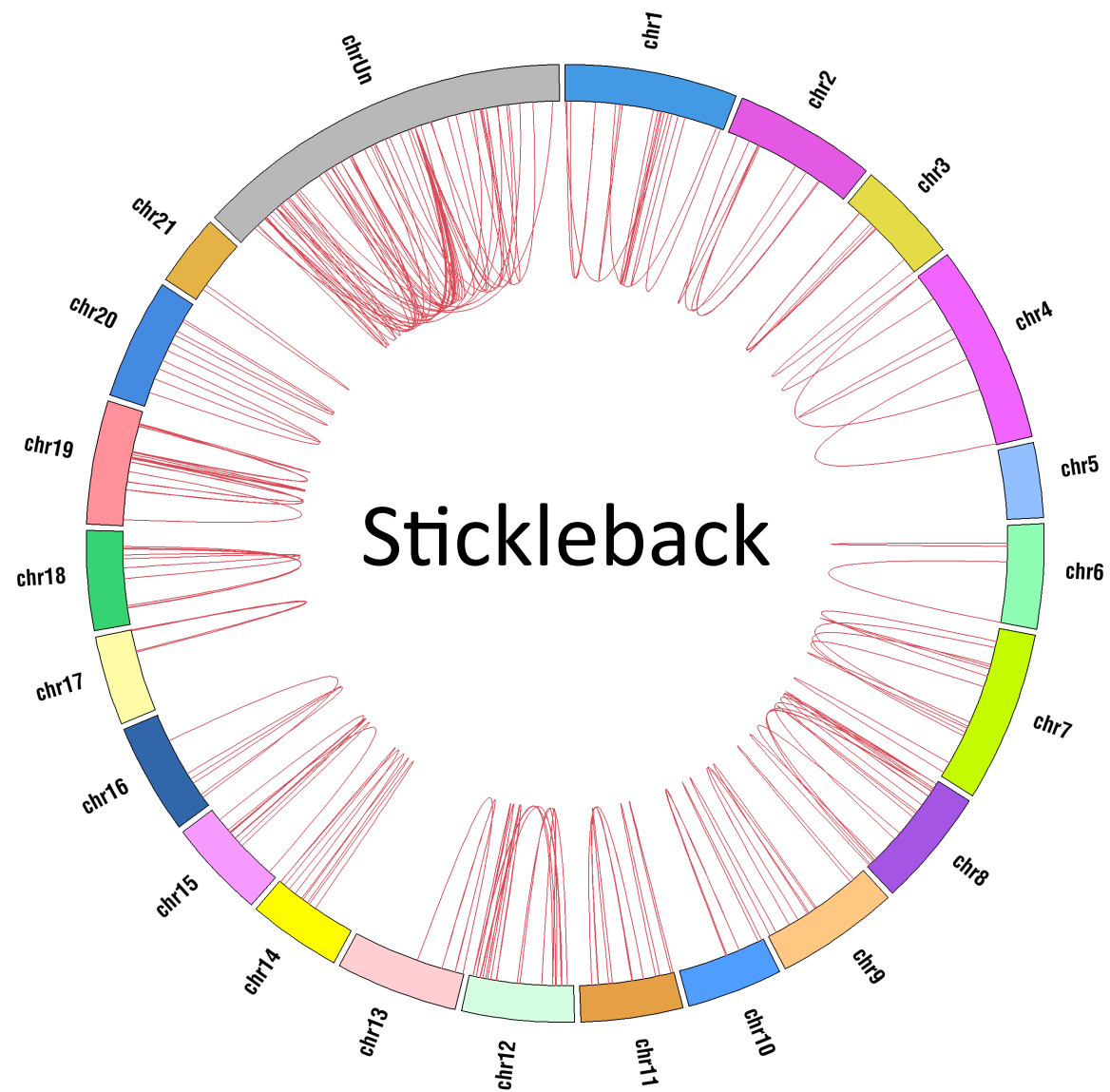

Supplement: Additional file 4 — Figure S1 Circos plot of the type 3 BLAST hits on the stickleback genome. The chromosomes of the stickleback genome are represented on the circle. The position of BAC mate pair BLAST hits are indicated with red arcs. [file 1471-2164-11-636-S4.PDF]

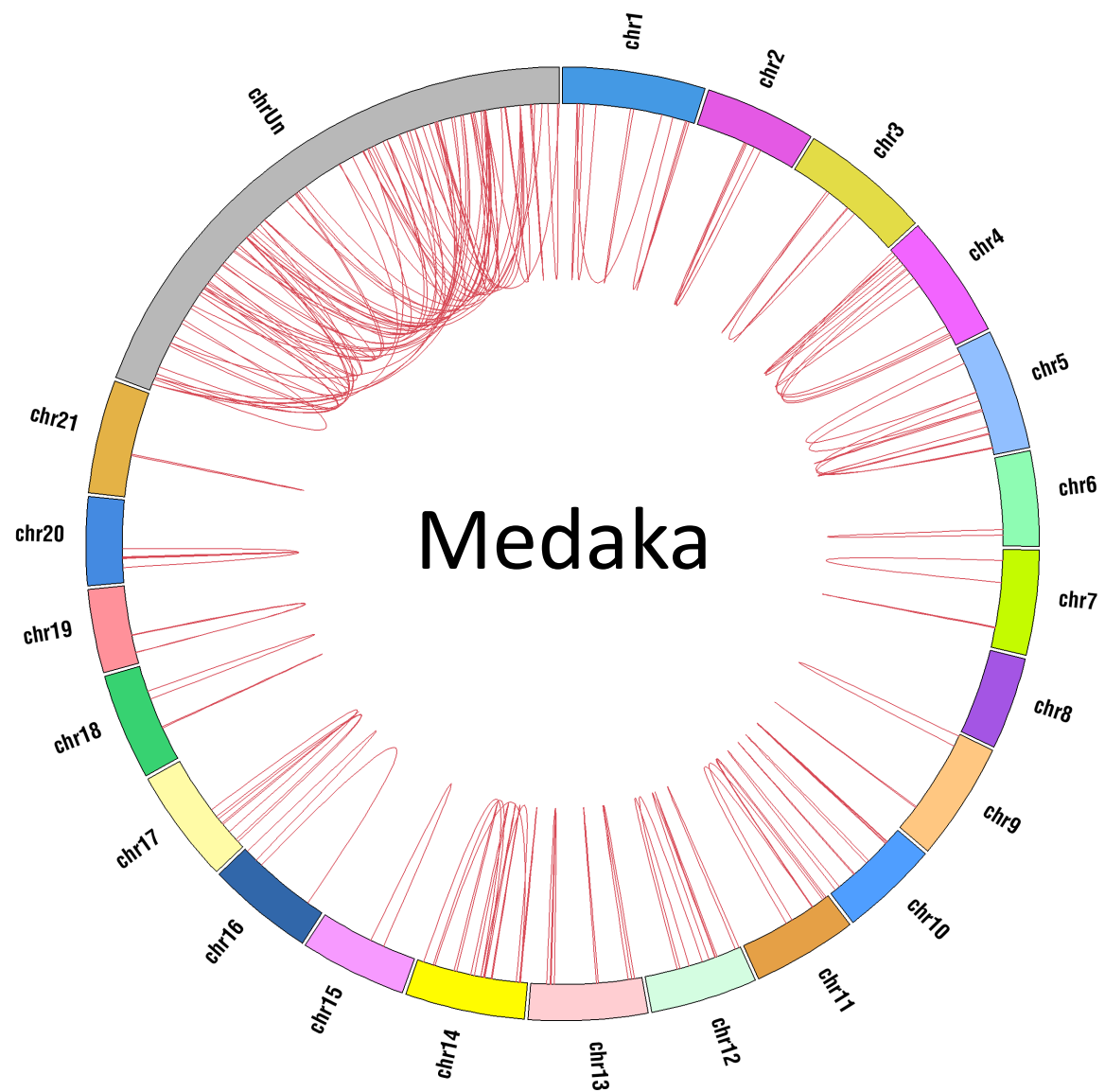

Supplement: Additional file 5 — Figure S2 Circos plot of the type 3 BLAST hits on the medaka genome. The chromosomes of the medaka genome are represented on the circle. The position of BAC mate pair BLAST hits are indicated with red arcs. [file 1471-2164-11-636-S5.PDF]

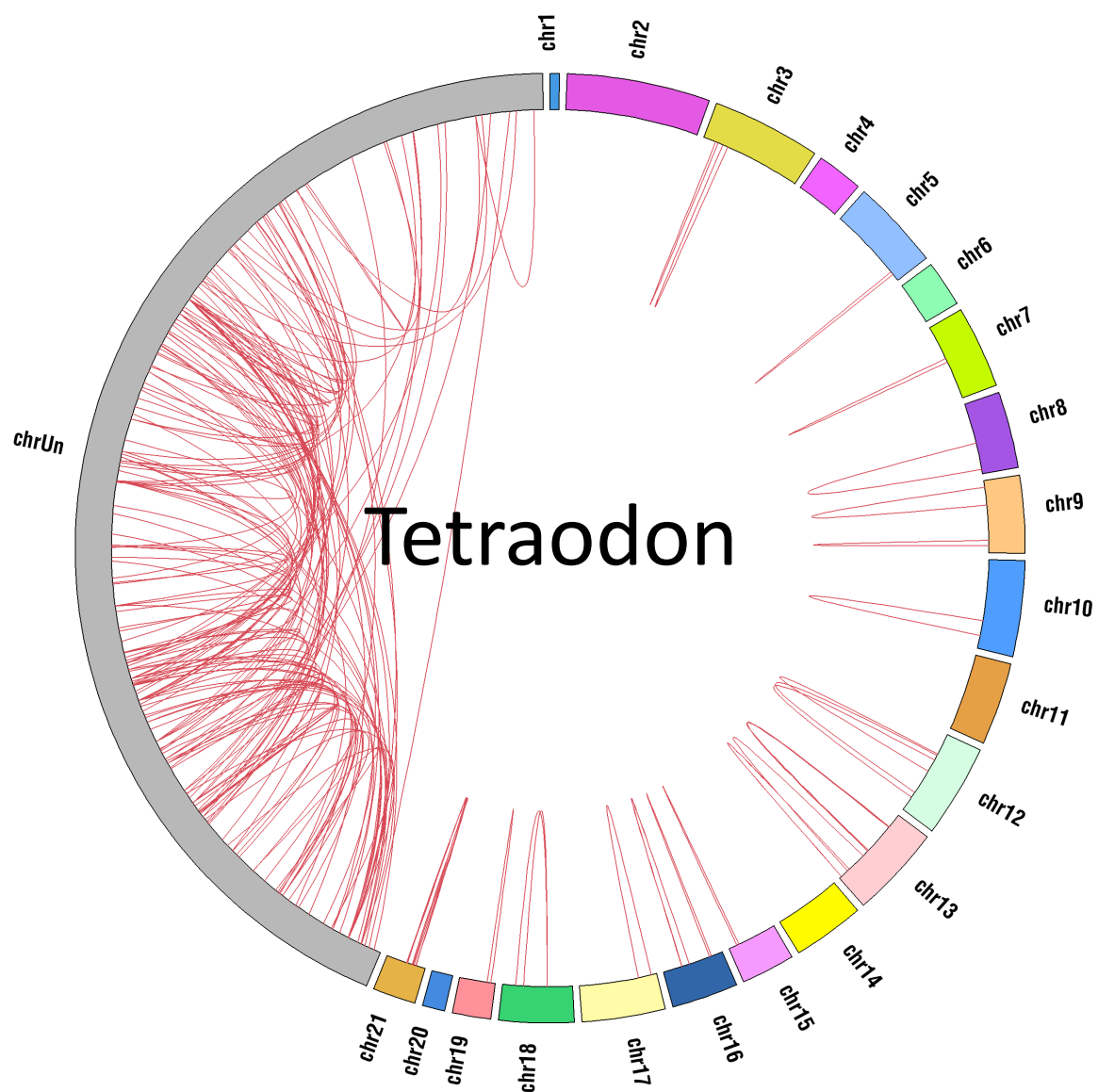

Supplement: Additional file 6 — Figure S3 Circos plot of the type 3 BLAST hits on the Tetraodon genome. The chromosomes of the Tetraodon genome are represented on the circle. The position of BAC mate pair BLAST hits are indicated with red arcs. [file 1471-2164-11-636-S6.PDF]
